# Effects of Collagen- and Arginine-Fortified Osteokine Supplementation on Fracture Healing

**DOI:** 10.7759/cureus.19072

**Published:** 2021-10-27

**Authors:** Kayahan Karaytug, Ufuk Arzu, Omer N Ergin, Fuat Bilgili, Gökcen Unverengil, Serkan Bayram, Cengiz Sen

**Affiliations:** 1 Orthopaedics, Acibadem Maslak Hospital, Istanbul, TUR; 2 Orthopaedics and Traumatology, Vehbi Koç Foundation (VKV) American Hospital, Istanbul, TUR; 3 Orthopaedics and Traumatology, Istanbul University Faculty of Medicine, Istanbul, TUR; 4 Pathology and Laboratory Medicine, Istanbul University Faculty of Medicine, Istanbul, TUR

**Keywords:** fracture, nutritional supplement, fracture healing, arginine, collagen

## Abstract

Introduction

Delayed union or nonunion is an important clinical challenge for orthopedic surgeons. In addition to the main treatment algorithms, the use of nutritional supplements is increasingly common. In this study, we investigated the effects of nutritional supplements fortified with arginine and collagen on fracture healing.

Materials and methods

Twenty-four rats with femur fractures were divided into experimental and control groups. Intramedullary fixation was performed in both groups. 20 ml/kg nutritional supplement was given to the experimental group. Radiological examination was performed at third and sixth weeks, and histopathological examination was performed at the sixth week.

Results

No statistically significant difference was found between the radiological scores of the groups at the third and sixth weeks. Nutritional supplement affected the histological properties of callus. Histological evidence of bone healing was observed by the sixth week in both groups but the score was higher in nutritional supplement group. A statistically significant difference was found between the histopathological scores of the groups at the sixth week.

Conclusion

Arginine- and type two collagen-augmented traditional nutritional supplements may help to achieve more successful results in fracture healing.

## Introduction

Due to its vascularity, bone tissue may be capable of self-healing [1,2]. Despite this outstanding healing capacity, bone union problems are seen in approximately 10% of fractures [1,3]. Delayed union and nonunion pose significant clinical challenges for orthopedic surgeons. The main strategies for the treatment of delayed union and nonunion include providing optimal mechanical stability and invasive supportive interventions such as growth factors, osteoconductive scaffolds, and stem cells [2,4]. Nutritional supplementation may also support bone healing. However, because the bone repair mechanism is complex, the non-invasive treatment methods and supplements that are widely used in recent times have not shown satisfactory effects [5,6].

Osteoblasts derived from mesenchymal stem cells (MSCs) play an important role in bone healing and remodeling. The transformation of pluripotent MSCs into osteoblasts with a high osteogenic capacity is important for increasing the success of remodeling and bone healing [7, 8].

Supplementation with osteokine, a natural compound used in traditional Chinese medicine, has been found to be effective in improving bone metabolism [5,6]. Osteokine is extracted from herbs that have been used to treat bone diseases in China for millennia [6]. Its formula is as follows: Chenpi (Citri Reticulatae Pericarpium), Hong Hua (*Carthami flos*), Sanqi (*Panax notoginseng*), Du Zhong (*Eucommia ulmoides*), Ren Shen (Panax ginseng), Huangqi (*Radix Astragali Mongolici*), and Bie Jia (*Carapax Trionycis*). It regulates renal and splenic activity to nourish the bone and produce multiple effects on bone repair and the expression of various genes related to bone formation and angiogenesis [5,9]. Thus, osteokine is clinically used to treat various orthopedic conditions, including femoral head necrosis, lumbar intervertebral disc prolapse, fracture, and osteoarthritis [6,10].

Osteokine® Plus (Crystal Natural Pharmaceutical Co. Ltd. Yunnan, China) is a commercial preparation of osteokine fortified with L-arginine, unmodified type II collagen, and hyaluronic acid. Although experimental and clinical studies have demonstrated the usefulness of osteokine in fracture healing, no study has investigated the effects of Osteokine Plus.

Here, we investigated the histopathological and radiological effects of Osteokine Plus on the healing of experimentally induced femur fractures in rats.

## Materials and methods

This experimental study included a total of 24 male Sprague-Dawley rats (Istanbul University, Institute of Experimental Medicine) with a mean age of 14 weeks and a mean weight of 250-300 g. The study was conducted according to the applicable institutional and national guidelines for the care and use of animals. The rats were randomly classified into the Osteokine Plus and control groups, each of which included 12 rats.

Surgical technique

All rats received an open fracture of the femoral diaphysis, which was fixed with an intramedullary Kirschner wire. An intraperitoneal injection of 65 mg/kg ketamine and 7 mg/kg xylazine was administered to all the animals for surgical anesthesia. Under sterile conditions, we exposed the femoral mid-diaphysis using a lateral approach, drilled through one-third of the diaphyseal diameter, and manually broke the remaining bone. We opened the knee with a 1 cm medial parapatellar incision and laterally dislocated the patella. After reducing the fracture without damaging the periosteum, we inserted a 21-gauge needle into the intramedullary canal through the femoral intercondylar notch and drilled the femur to achieve the most stable fixation possible. A Kirschner wire (diameter: 0.71-1.25 mm) was advanced proximally to the greater trochanter, and the distal end of the wire was cut and positioned inside the intercondylar notch. In all the rats, the incisions were closed with 5-0 vicryl sutures.

Treatment and follow-up

For postoperative pain control, 0.2 mg/kg morphine sulfate end of the surgery and twice on the postoperative first day was administered subcutaneously. The animals were evaluated twice a day for swelling, complications, nutrition, and hydration. Postoperatively, the rats were fed a standard diet and water. For analgesia, carprofen (4 mg/kg; Rimadyl, Pfizer, New York City, NY) was subcutaneously administered once a day for three days. To prevent infection, ceftriaxone (25 mg/kg; Rocephin, Roche, Basel, Switzerland) was intramuscularly injected once a day for three days.

Each rat in the experimental group received a daily dose of 20 mL/kg Osteokine Plus (Crystal Natural Pharmaceutical Co. Ltd. Yunnan, China). The compound was systemically administered through a nasogastric tube, starting from the day of the surgery to the day the animals were sacrificed. The rats in the control group did not receive Osteokine Plus treatment.

All rats in both the control and Osteokine Plus group were followed for six weeks. At the end of the study period, all the animals were euthanized by intraperitoneal injection of sodium pentothal (135 mg/kg). All the procedures were performed using aseptic techniques.

Radiological evaluation

Using a digital imaging system, radiographs were obtained for all rats at three and six weeks after surgery. The radiographs were taken under anesthesia with the rats in the prone position with both hindlimbs abducted. Callus formation and osseous union were evaluated using the Lane-Sandhu system, in which fracture healing is graded as 0 (no callus), 1 (minimal callus), 2 (callus evident but healing incomplete), 3 (callus evident with stability expected), or 4 (complete healing with bone remodeling) [11].

Histological evaluation

After implant removal, the femurs were removed, fixed in 10% formalin for one day, and subsequently decalcified in a 10% nitric acid solution for two days. The tissue samples were processed and embedded in paraffin. The sections were stained with hematoxylin-eosin and histopathologically examined. Inflammation and callus formation were graded using Huo histopathological scoring system that describes callus formation from fibrous tissue to bone healing with mature bone [12].

Statistical analyses

Number Cruncher Statistical System (NCSS) software was used for statistical analysis. Descriptive statistical methods (mean, standard deviation, median, frequency, and ratio) were used to evaluate the data, and the Mann-Whitney U test was used for the intergroup comparison of parameters that were not normally distributed. The level of significance was set at p < 0.05.

## Results

**Figure 1:**
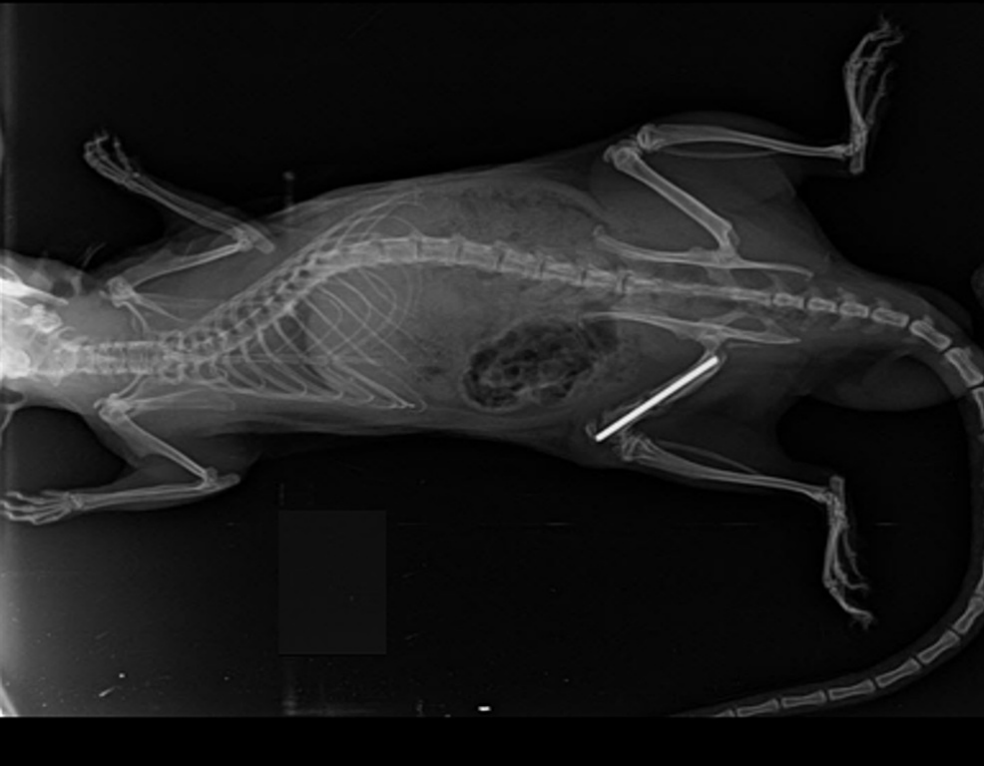
In the third week radiograph of a rat in the control group In the third week radiograph of a rat belonging to the control group, it was observed that callus formation and bone union started.

**Figure 2:**
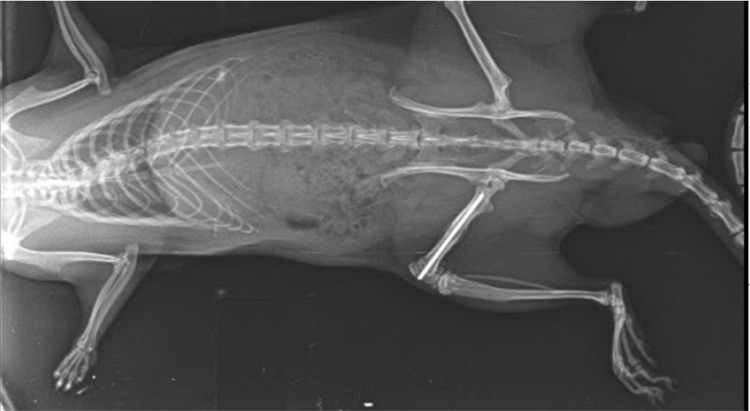
Complete bone union in the sixth week in Osteokine Plus group Complete bone union was observed in the sixth week radiograph of a rat belonging to the osteokine plus group.

Osteokine Plus did not significantly alter the radiographic appearance of the callus. Radiological evidence of fracture healing (Lane-Sandhu score of 1-2) was observed at three weeks postoperatively (Figure 1), and almost complete union (Lane-Sandhu score of 3-4) was observed at six weeks postoperatively in both the Osteokine Plus and control groups (Figure 2).

No statistically significant between-group difference in radiological score was found at three and six weeks postoperatively (p > 0.05). The rate of increase in radiological score was the same at three and six weeks postoperatively in both groups, and there was no significant difference between the two groups (p > 0.05; Table 1).

**Table 1:** Evaluation of the radiological scores according to groups

Radiological score		Osteokine Plus group (n)	Control Group (n)	P
Week 3	Score 1	5 (41.7%)	4 (33.3%)	
	Score 2	7 (58.3%)	8 (66.7%)	
	Median (Min-Max)	2 (1-2)	2 (1-2)	0.755
Week 6	Score 3	4 (33.3%)	6 (50.0%)	
	Score 4	8 (66.7%)	6 (50.0%)	
	Median (Min-Max)	4 (3-4)	3.5 (3-4)	0.514
Radiological score differences Δ	Median (Min-Max)	2 (1-3)	2 (1-3)	0.378

**Figure 3:**
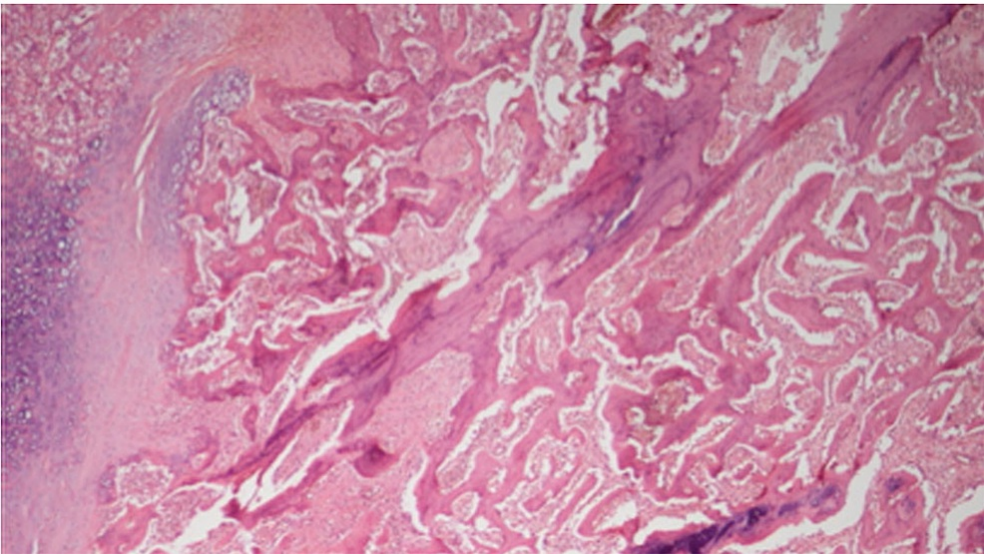
Immature bone and focally of cartilage in callus tissue The control group at six weeks shows callus tissue, consisting predominantly of immature bone and focally of cartilage (Stain, hematoxylin-eosin; original magnification, x100)

Osteokine Plus affected the histological properties of the callus. Histological evidence of bone healing was observed by the sixth week in both groups, but the Osteokine Plus group had a higher Huo classification score [12]. All tissue samples showed areas of polymorphonuclear leukocyte aggregation and callus formation. In the third week, areas of cartilage formation (Huo histopathological score 8 and 9: mostly immature bone with some cartilage tissue) were observed in the tissue samples of both groups (Figure 3) [12].

**Figure 4:**
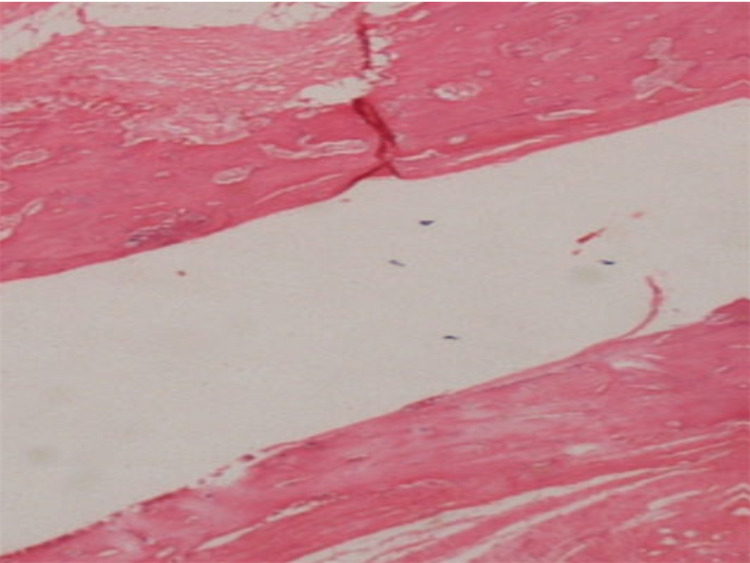
Cortical bridging area in callus One case in the Osteokine Plus group show callus tissue with bone, showing bridging in the cortical area (Stain, hematoxylin-eosin; original magnification, x40)

In addition, in the Osteokine Plus group, nearly mature bone tissue that showed trabecular formation was observed (Huo histopathological score 10: fracture healing with mature bone (Figure 4) [12].

A statistically significant between-group difference was found in the histopathological scores at six weeks postoperatively (p < 0.05; Table 2).

**Table 2:** Evaluation of histopathological scores according to groups * p<0.05

Week 6		Osteokine plus Group	Control Group	P
Histopathological Score	Mean+SD	9.33±0.78	8.58±0.51	0.024*
	Median (Min-Max)	9.5 (8-10)	9 (8-9)	

In the Osteokine Plus group, a statistically significant association was found between the histopathological and radiological scores (p < 0.05); as the radiological score increased, so did the histopathological score. In the control group, there was no statistically significant association between the radiological and histopathological scores (p > 0.05; Table 3).

**Table 3:** Relationship between radiological and histopathological scores at six weeks * p<0.05

Week 6	Radiological score		N	Histopathological score		P
				Median	Min-Max	
Osteokine Plus group		Score 3	4	9.00	8-9	0.041*
		Score 4	8	10.00	8-10	
Control group		Score 3	6	8.00	8-9	0.180
		Score 4	6	9.00	8-9	

## Discussion

Bone healing is a complex but well-organized physiological process in which hematoma formation, inflammation and repair, and remodeling controlled by growth factors occur, in that order [13-15]. It is estimated that 5-10% of all fractures are associated with bone healing problems such as malunion or nonunion, which poses a challenge for the surgeon. The causes of nonunion may be patient-dependent, patient-independent, mechanical, or biological [16,17]. Optimal biological environment and mechanical stability are vital for preventing nonunion. Biological healing is based on stimulation of the successful differentiation of MSCs into osteoblasts at the fracture site. Supplements are increasingly being used to successfully achieve this [18].

Osteokine is widely used as a supplement in traditional Chinese medicine. It has a positive effect on bone metabolism. Sun et al. showed that osteokine supplementation led to improved bone mass, trabecular number, and bone volume fraction [9]. Microscopic observation and micro-computed tomography revealed that osteokine supplementation led to good callus formation in patients with osteoporotic fractures [9,11]. The mechanism underlying the effects of osteokine on fracture healing is closely related to the differentiation of MSCs into osteoblasts [7,8,19,20]. Furthermore, osteokine compounds consist of components that could lead to upregulation of runt-related transcription factor 2 (RUNX2) and vascular endothelial growth factor (VEGF) expression, which may enhance bone formation and vascular regeneration [5,10]. Osteokine Plus is comprised of osteokine fortified with L-arginine, type II collagen, and hyaluronic acid.

L-arginine plays an active role in many physiological processes. It has been found to increase collagen accumulation and wound-breaking strength, play a role in anti-aging and antioxidant activities, regulate immunity, enhance the biological crosslinking of natural polymers, and stimulate MSC growth and differentiation [21-24]. Furthermore, type II collagen plays an important role in enchondral healing and soft callus formation [25]. The Lane-Sandhu radiological scores of the Osteokine Plus group were better than those of the control group, indicating that Osteokine Plus had a positive effect on fracture healing. Also, half of the rats in the control group showed histopathological findings of “predominantly immature bone and a small amount of cartilage tissue,” and “fracture healing with immature bone” was observed in the other half. In contrast, two-thirds of the rats in the Osteokine Plus group showed histopathological findings of “fracture healing with mature bone.” Consequently, Osteokine Plus can be considered to have improved fracture healing and mature bone formation. Therefore, Osteokine Plus, which is fortified with arginine and type II collagen, is thought to contribute to improved callus formation during fracture healing through fortification with these ingredients. However, histopathological examination was performed only in the area of callus formation, and cellular analysis and biomechanical evaluation were not performed, which may be the limitations of the study. In addition, the scope of the study could not be expanded because the ethics committee did not approve the inclusion of more rats, which was required to enable long-term follow-up and evaluate the effects of different doses.

## Conclusions

Osteokine Plus plays an active role in various physiological processes in the human body, and it contains compounds that act as modulators of the healing process. The addition of arginine and type II collagen to osteokine can improve its effect on fracture healing. We believe that Osteokine Plus, which can be recommended along with other treatment modalities, will help to improve fracture healing outcomes in patients with patient-related negative prognostic factors.
